# Predictive value of the monocyte-to-albumin ratio at admission for 30-day mortality in elderly patients with spontaneous intracerebral hemorrhage: a retrospective analysis

**DOI:** 10.3389/fneur.2026.1948936

**Published:** 2026-09-30

**Authors:** Lihui Gong, Fenglin Liu, Keyi Zhao, Haoshuang Zhao, Sujun Guo, Santao Zhang

**Affiliations:** Department of Neurosurgery, Shanxi Provincial People's Hospital, Taiyuan, Shanxi, China

**Keywords:** biomarker, elderly, inflammation, intracerebral hemorrhage, monocyte-to-albumin ratio, mortality, prognosis, risk stratification

## Abstract

**Background:**

The monocyte-to-albumin ratio (MAR) integrates systemic inflammation and nutritional status and has been proposed as a predictor of mortality after spontaneous intracerebral hemorrhage (sICH). However, independent validation in an exclusively elderly cohort remains limited. We aimed to evaluate the association between admission MAR and 30-day mortality in patients aged ≥65 years with sICH.

**Methods:**

This single-center retrospective study included 206 consecutive elderly patients with acute sICH. MAR was calculated as monocyte count (×10^9^/L) divided by serum albumin (g/L). The primary outcome was 30-day all-cause mortality. Optimal MAR cutoff was determined by Youden’s index. Kaplan–Meier and multivariable Cox regression analyses were performed.

**Results:**

30-day mortality was 38.8%. MAR was significantly higher in non-survivors (0.022 vs. 0.016, *p* < 0.001). The optimal cutoff was 0.021 (AUC 0.648, 95% CI 0.572–0.724), providing sensitivity of 57.5% and specificity of 68.3%. MAR performance was comparable to the neutrophil-to-lymphocyte ratio (AUC 0.643) and systemic inflammatory response index (AUC 0.637). After adjustment, MAR > 0.021 was independently associated with 30-day mortality (adjusted HR 2.41, 95% CI 1.51–3.85, *p* < 0.001). In a sensitivity analysis using the ICH score as the primary severity covariate, MAR remained independently associated with mortality (adjusted HR 2.02, 95% CI 1.25–3.26, *p* = 0.004).

**Conclusion:**

Admission MAR is independently associated with 30-day mortality in elderly sICH patients, although its discriminative ability is limited (AUC 0.648). As a simple, routinely available parameter, MAR may serve as a supplementary—not standalone—bedside risk stratification tool.

## 1 Introduction

Spontaneous intracerebral hemorrhage (sICH) is the most devastating stroke subtype, accounting for 10–20% of all strokes but carrying a 30-day mortality rate of 30–50% (1, 2). The prognosis is particularly poor in patients aged 65 years and older, in whom age-related immune dysregulation, pre-existing malnutrition, and frailty synergistically amplify secondary brain injury and restrict neurological recovery (3). Early identification of high-risk patients is essential for guiding clinical decision-making, allocating monitoring resources, and initiating timely nursing interventions.

The systemic inflammatory response and nutritional status are two closely intertwined determinants of outcome after sICH. Monocytes infiltrate the perihematomal region and contribute to blood–brain barrier disruption, vasogenic edema, and neuronal apoptosis through the release of pro-inflammatory cytokines such as tumor necrosis factor-α and interleukin-1β (4). Hypoalbuminemia, highly prevalent in the elderly, reflects protein-energy malnutrition and a diminished capacity to counteract oxidative stress, and has been independently associated with larger hematoma volumes and worse outcomes in hemorrhagic stroke (5, 6).

The monocyte-to-albumin ratio (MAR) integrates these two pathophysiological pathways into a single accessible index. In 2026, Jaradat et al. published a landmark study in PLoS One demonstrating that an admission MAR > 0.018 independently predicted 30-day and 90-day mortality in 118 sICH patients with a mean age of 74 years (adjusted odds ratio 3.57; area under the curve [AUC] 0.606) (7). This finding established MAR as a promising prognostic biomarker and called for external validation in independent cohorts. Subsequent research by Fu et al. reported that MAR was also associated with hematoma expansion in sICH, further supporting its pathophysiological relevance (8).

Beyond MAR, several other composite inflammatory-nutritional indices have been investigated in sICH. The neutrophil-to-albumin ratio (NAR) has emerged as a predictor of mortality in a multicenter retrospective cohort study (9). The lymphocyte-to-monocyte ratio (LMR), reflecting the balance between adaptive and innate immunity, has shown prognostic value in sICH, with slightly elevated LMR predicting favorable outcomes (10). The glucose-to-albumin ratio has also been proposed as a novel biomarker for mortality prediction after ICH (11). The C-reactive protein-to-albumin ratio, which similarly integrates inflammation and nutrition, has been shown to predict early mortality in hospitalized older patients independent of admission diagnosis (12). The neutrophil-to-lymphocyte ratio (NLR) and systemic inflammatory response index (SIRI) are the most extensively studied inflammatory indices in sICH, with meta-analytic evidence supporting their association with mortality and functional outcomes (13). However, few studies have directly compared the predictive performance of MAR with these established indices specifically in an elderly sICH population.

Therefore, we conducted this retrospective study with the following objectives: (1) to independently validate the association between admission MAR and 30-day all-cause mortality in an elderly sICH cohort; (2) to compare the discriminative ability of MAR with NLR and SIRI; and (3) to discuss the clinical feasibility of MAR as a simple, nurse-friendly supplementary tool for bedside risk stratification.

## 2 Materials and methods

### 2.1 Study design and setting

This was a single-center retrospective observational cohort study conducted at the Department of Neurosurgery, Heping Campus, Shanxi Provincial People’s Hospital, a university-affiliated tertiary medical center and regional stroke center in Taiyuan, Shanxi Province, China. The study was approved by the Ethics Committee of Shanxi Provincial People’s Hospital (Approval No. (2026) Provincial Medical Ethics Review No. 848). The requirement for written informed consent was waived owing to the retrospective design.

### 2.2 Study population

Consecutive patients aged ≥65 years admitted with a primary diagnosis of acute sICH between 1 January 2025 and 31 May 2026 were screened. Diagnosis was confirmed by non-contrast head computed tomography (CT) within 6 h of symptom onset. Exclusion criteria were: (1) secondary ICH due to trauma, aneurysm rupture, arteriovenous malformation, brain tumor, hemorrhagic transformation of ischemic stroke, severe coagulation factor deficiency (e.g., hemophilia), or severe thrombocytopenia (platelet count <50 × 10^9^/L); (2) use of immunosuppressive agents, corticosteroids (≥10 mg/day prednisone equivalent), chemotherapy, or biologics within 4 weeks before admission; (3) active infection (procalcitonin >0.5 ng/mL or positive blood culture); (4) end-stage liver disease (Child-Pugh class C with ascites, encephalopathy, or jaundice), end-stage renal disease (estimated glomerular filtration rate <15 mL/min/1.73 m^2^ or maintenance dialysis), or active malignancy (defined as diagnosis within 5 years, ongoing or planned palliative treatment, or expected survival <6 months; patients with curatively treated cancers and no recurrence for >5 years were eligible); and (5) missing admission complete blood count or albumin data; and (6) death within 24 h of admission. After screening 268 patients, 206 were included (Supplementary Figure S1).

### 2.3 Data collection

Data were independently extracted from electronic medical records by two investigators. Demographics, medical history, admission Glasgow Coma Scale (GCS) score, and the ICH score (14) were collected. Hematoma volume was measured using the ABC/2 method by a neuroradiologist blinded to outcomes. Intraventricular hemorrhage (IVH) and infratentorial location were recorded. Blood samples were obtained within 30 min of emergency department arrival. Complete blood count with differential was performed on a Sysmex XN-9000 analyzer. Serum albumin was measured by the bromocresol green method on a Beckman Coulter AU5800 analyzer. MAR was defined as monocyte count (×10^9^/L) divided by albumin (g/L); NLR as neutrophil count divided by lymphocyte count; and SIRI as (neutrophil count × monocyte count) divided by lymphocyte count.

### 2.4 Outcomes

The primary outcome was 30-day all-cause mortality, ascertained from hospital records, telephone follow-up, and the provincial death registry. Secondary outcomes were 48-h mortality, 90-day mortality, and hospital length of stay among survivors. No patients were lost to 90-day follow-up. Functional outcome at 90 days (modified Rankin Scale, mRS) was not collected in the original dataset and therefore could not be analyzed.

### 2.5 Statistical analysis

Continuous variables are presented as median (interquartile range [IQR]) and compared using the Mann–Whitney *U* test. Categorical variables are presented as frequency (%) and compared with the Chi-square test. Receiver operating characteristic (ROC) curves were constructed for MAR, NLR, and SIRI. The optimal MAR cutoff was determined by maximizing Youden’s index. AUCs were compared using the DeLong test. Kaplan–Meier curves were compared with the log-rank test. Cox proportional hazards regression estimated adjusted hazard ratios (HRs) with 95% confidence intervals (CIs). The primary multivariable model included variables selected based on clinical relevance: age, GCS, hematoma volume, and IVH. Given structural collinearity between GCS and the ICH score (variance inflation factor [VIF] > 5), the ICH score was excluded from the primary multivariable model. As a sensitivity analysis, a separate model was constructed with the ICH score (continuous) as the primary severity covariate, replacing GCS and IVH, which are components of the ICH score. The proportional hazards assumption was verified with Schoenfeld residuals for each covariate. No missing data existed for the variables used in the primary model. Sensitivity analyses excluded patients who died between 24 and 48 h and included the ICH score as a continuous variable. No formal sample size calculation was performed. Two-tailed *p* < 0.05 was considered significant. Analyses used SPSS 26.0 (IBM Corp., Armonk, NY, USA) and R 4.2.3 (R Foundation, Vienna, Austria). Net Reclassification Improvement (NRI), Integrated Discrimination Improvement (IDI), calibration assessment, and decision curve analysis (DCA) could not be performed because individual-level predicted probabilities were not retained in the original dataset.

## 3 Results

### 3.1 Patient disposition and baseline characteristics

Of 268 patients screened, 62 were excluded (Supplementary Figure S1): 18 with secondary ICH, 14 with active infection, 10 with immunosuppressant use, 8 with end-stage organ disease or active malignancy, 8 who died within 24 h of admission, and 4 with missing data. The final cohort comprised 206 patients. The 30-day mortality rate was 38.8% (80/206). The 48-h mortality rate was 5.3% (11/206). Among these 80 deaths, 11 occurred between 24 and 48 h after admission, and 69 occurred between 48 h and 30 days. Non-survivors had a significantly higher median MAR than survivors (0.022 vs. 0.016, *p* < 0.001) and higher NLR and SIRI (Table 1).

**Table 1:** Baseline characteristics of elderly sICH patients by 30-day survival status.

Characteristic	All patients (*n* = 206)	Survivors (*n* = 126)	Non-survivors (*n* = 80)	*p*-value
Demographics				
Age, years, median (IQR)	74 (68–80)	72 (67–77)	77 (71–83)	<0.001
Male sex, *n* (%)	110 (53.4)	70 (55.6)	40 (50.0)	0.478
Medical history, *n* (%)				
Hypertension	168 (81.6)	102 (81.0)	66 (82.5)	0.781
Diabetes mellitus	58 (28.2)	34 (27.0)	24 (30.0)	0.638
Atrial fibrillation	24 (11.7)	13 (10.3)	11 (13.8)	0.453
Prior stroke	50 (24.3)	28 (22.2)	22 (27.5)	0.387
Clinical severity				
GCS score, median (IQR)	13 (8–15)	14 (13–15)	8 (5–11)	<0.001
Hematoma volume, mL, median (IQR)	24.5 (11.0–48.8)	16.0 (8.5–28.0)	48.0 (31.0–72.0)	<0.001
IVH, *n* (%)	54 (26.2)	20 (15.9)	34 (42.5)	<0.001
Infratentorial, *n* (%)	14 (6.8)	4 (3.2)	10 (12.5)	0.012
ICH score, median (IQR)	1 (1–2)	1 (0–1)	2 (1–3)	<0.001
Laboratory findings				
Monocyte, ×10^9^/L, median (IQR)	0.56 (0.42–0.82)	0.52 (0.38–0.69)	0.76 (0.54–1.05)	<0.001
Albumin, g/L, median (IQR)	35.8 (30.5–39.9)	38.0 (34.5–41.5)	31.0 (27.2–35.8)	<0.001
MAR, median (IQR)	0.017 (0.013–0.026)	0.016 (0.011–0.020)	0.022 (0.017–0.034)	<0.001
NLR, median (IQR)	4.60 (2.80–7.70)	3.65 (2.40–5.60)	6.50 (4.15–10.85)	<0.001
SIRI, median (IQR)	2.75 (1.38–5.75)	1.90 (1.02–3.45)	5.20 (2.80–10.30)	<0.001

IQR, interquartile range; GCS, Glasgow Coma Scale; IVH, intraventricular hemorrhage; MAR, monocyte-to-albumin ratio; NLR, neutrophil-to-lymphocyte ratio; SIRI, systemic inflammatory response index.

### 3.2 ROC analysis and optimal MAR cutoff

MAR predicted 30-day mortality with an AUC of 0.648 (95% CI 0.572–0.724, *p* < 0.001; Figure 1). Table 2 shows the candidate cutoffs. The maximum Youden index was 0.259 at a cutoff of 0.022, with a nearly identical value of 0.258 at 0.021. We selected 0.021 as the optimal threshold because it offers a clinically more acceptable balance—sensitivity of 57.5% vs. 51.3% at 0.022—thereby reducing the number of high-risk patients who would be missed, while still maintaining reasonable specificity (68.3%). The AUC for NLR was 0.643 (95% CI 0.566–0.720) and for SIRI was 0.637 (95% CI 0.559–0.715); DeLong tests showed no significant differences (MAR vs. NLR, *p* = 0.893; MAR vs. SIRI, *p* = 0.798; Figure 2).

**Figure 1:**
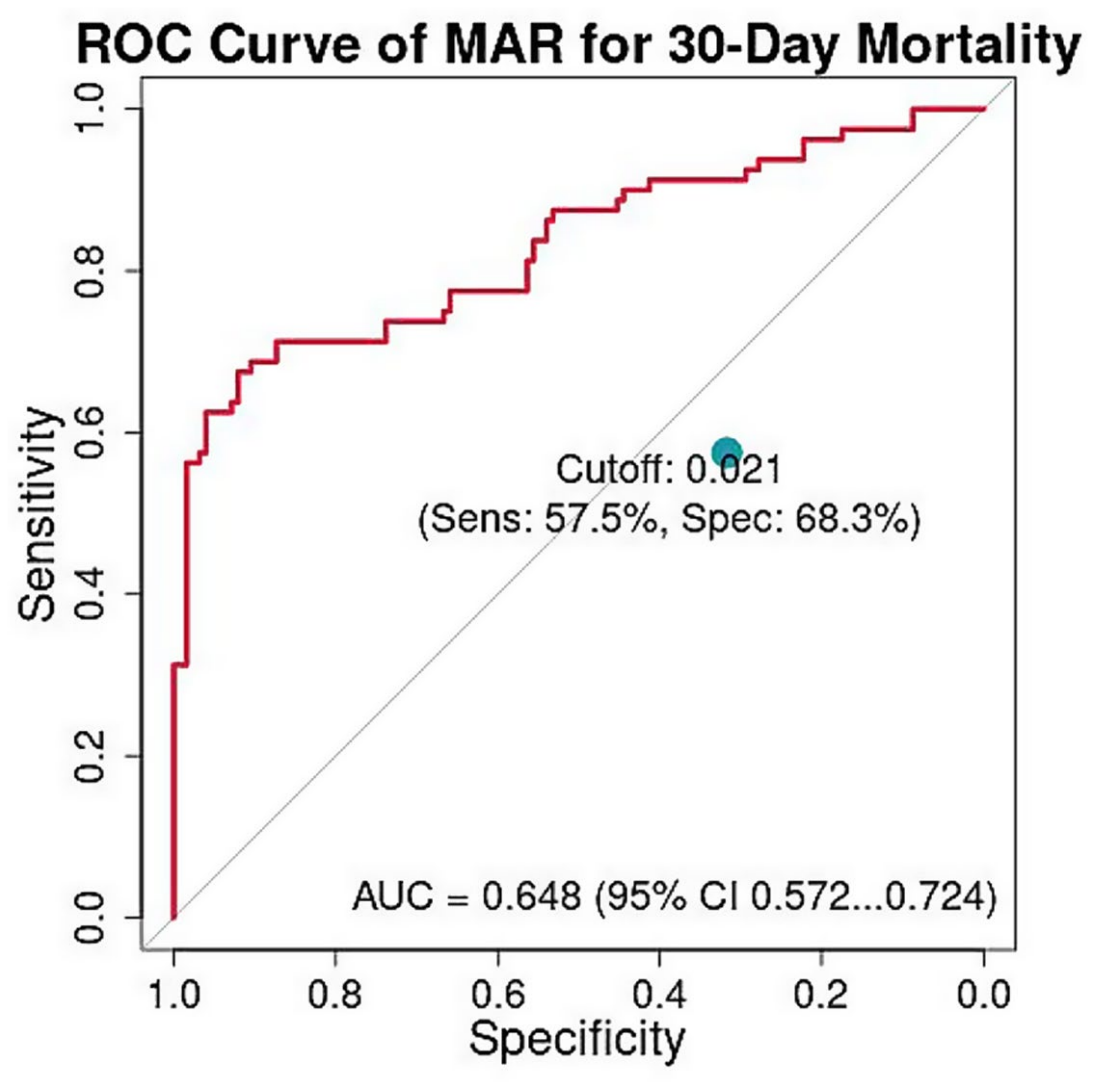
Receiver operating characteristic (ROC) curve of admission MAR for predicting 30-day mortality in elderly sICH patients. AUC = 0.648 (95% CI 0.572–0.724). The optimal cutoff (0.021), determined by Youden’s index, is indicated by the blue filled circle.

**Table 2:** Youden index analysis for MAR in predicting 30-day mortality.

MAR cutoff	Sensitivity (%)	Specificity (%)	Youden index
0.018	67.5	50.8	0.183
0.019	63.8	57.9	0.217
0.020	61.3	63.5	0.248
0.021	57.5	68.3	0.258
0.022	51.3	74.6	0.259
0.023	45.0	79.4	0.244

**Figure 2:**
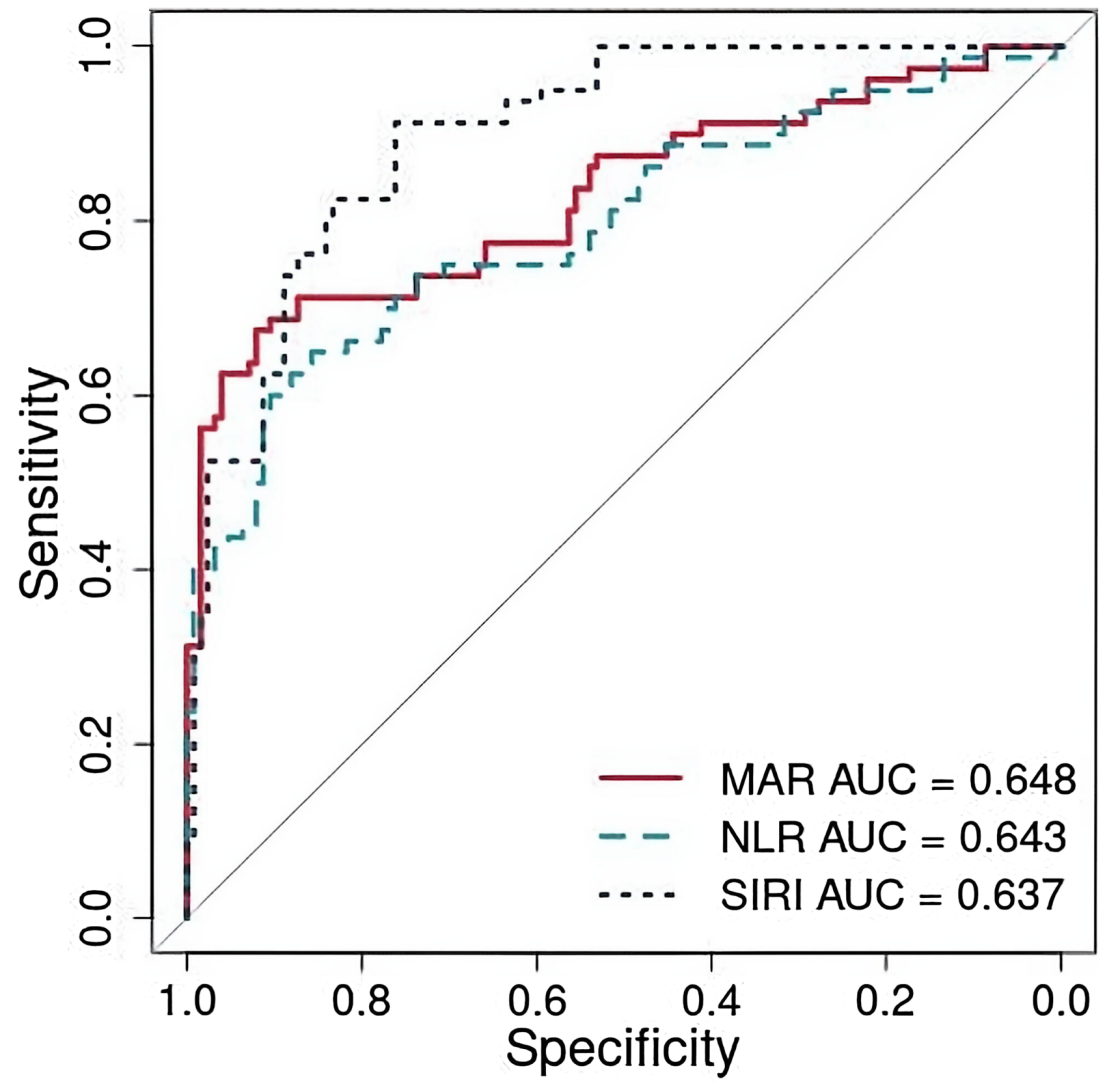
Comparative ROC curves for MAR, NLR, and SIRI. AUC values: MAR 0.648, NLR 0.643, SIRI 0.637. No significant differences among the three indices by DeLong test (MAR vs. NLR, *p* = 0.893; MAR vs. SIRI, *p* = 0.798).

The optimal cutoff was set at 0.021 to balance sensitivity and specificity; although the Youden index at 0.022 is marginally higher (0.259 vs. 0.258), adopting 0.022 would have reduced sensitivity by 6.2 absolute percentage points, potentially missing more high-risk patients.

### 3.3 Kaplan–Meier survival analysis

Kaplan–Meier curves showed significantly higher 30-day mortality in patients with MAR > 0.021 compared to those with MAR ≤ 0.021 (53.5% vs. 28.3%; log-rank *χ*^2^ = 17.2, *p* < 0.001; Figure 3). The groups comprised 86 and 120 patients, respectively, consistent with the sensitivity and specificity reported in Table 2. The baseline characteristics of these two groups are compared in Supplementary Table S1.

**Figure 3:**
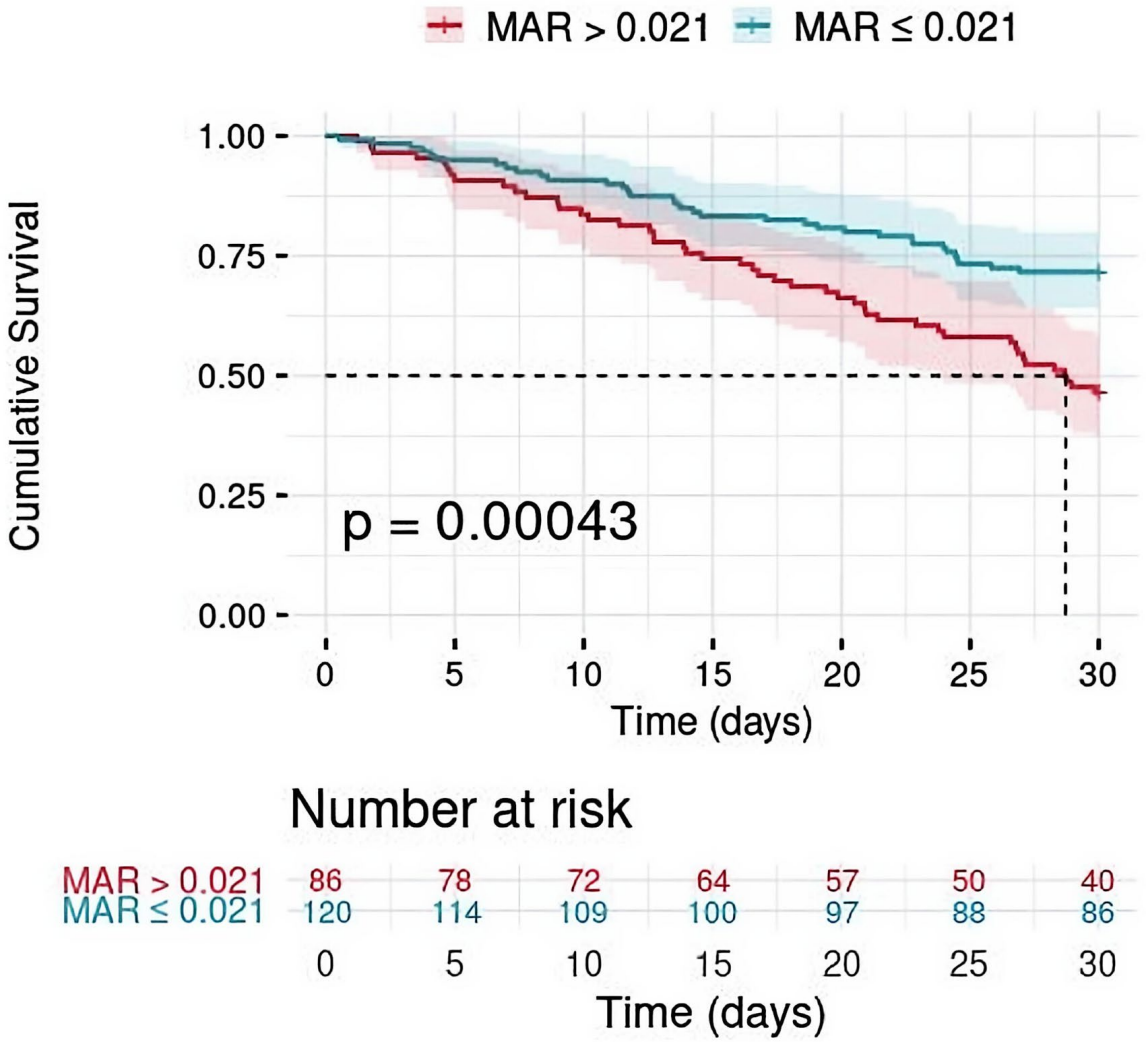
Kaplan–Meier 30-day survival curves stratified by MAR > 0.021 (*n* = 86) versus MAR ≤ 0.021 (*n* = 120). The cumulative 30-day mortality was 53.5% in the high-MAR group and 28.3% in the low-MAR group (log-rank *χ*^2^ = 17.2, *p* < 0.001). Shaded areas represent pointwise 95% confidence intervals.

### 3.4 Multivariable cox regression

Collinearity between GCS and ICH score was confirmed (VIF = 6.9); thus, the ICH score was excluded from the primary model. After adjustment, MAR > 0.021 was independently associated with 30-day mortality (adjusted HR 2.41, 95% CI 1.51–3.85, *p* < 0.001; Table 3). GCS (adjusted HR 0.87, 95% CI 0.81–0.93, *p* < 0.001) and hematoma volume (adjusted HR 1.02, 95% CI 1.01–1.03, *p* < 0.001) were also independently significant.

**Table 3:** Multivariable cox regression for 30-day mortality (primary model).

Variable	Crude HR (95% CI)	*p*-value	Adjusted HR (95% CI)	*p*-value
MAR > 0.021	2.63 (1.67–4.15)	<0.001	2.41 (1.51–3.85)	<0.001
Age, per year	1.05 (1.02–1.09)	0.002	1.02 (0.99–1.05)	0.217
GCS, per point	0.79 (0.74–0.84)	<0.001	0.87 (0.81–0.93)	<0.001
Hematoma volume, per mL	1.03 (1.02–1.04)	<0.001	1.02 (1.01–1.03)	<0.001
IVH, yes vs. no	2.92 (1.84–4.63)	<0.001	1.18 (0.72–1.94)	0.513

To address the concern that the ICH score is the most widely validated prognostic scale, we performed a sensitivity analysis in which the ICH score (continuous) replaced GCS and IVH as the primary severity covariate. In this model, MAR > 0.021 remained independently associated with 30-day mortality (adjusted HR 2.02, 95% CI 1.25–3.26, *p* = 0.004; Supplementary Table S2). The C-statistic for the ICH score alone was 0.731; adding MAR increased it to 0.751 (Δ*C* = 0.020).

### 3.5 MAR and baseline hematoma volume

Linear regression showed a weak positive association between MAR and hematoma volume (standardized *β* = 0.20, *p* = 0.004; Spearman *ρ* = 0.23, *p* < 0.001; Supplementary Figure S2). This finding is consistent with prior work demonstrating a relationship between MAR and hematoma expansion (8).

### 3.6 Sensitivity and secondary analyses

After excluding 11 patients who died between 24 and 48 h after admission, MAR > 0.021 remained a significant predictor (adjusted HR 2.18, 95% CI 1.29–3.68, *p* = 0.003). The full multivariable model for this sensitivity analysis is provided in Supplementary Table S3. MAR > 0.021 was also associated with 90-day mortality (unadjusted log-rank *p* < 0.001; adjusted HR 2.05, 95% CI 1.33–3.16, *p* = 0.001). The complete predictive performance metrics for MAR, NLR, and SIRI at 90 days are presented in Supplementary Table S4. Hospital length of stay among survivors could not be reported because the original dataset did not include this variable in analyzable form.

## 4 Discussion

In this independent, single-center retrospective cohort of 206 elderly patients with acute sICH, admission MAR > 0.021 was independently associated with 30-day all-cause mortality after adjustment for GCS score and hematoma volume. The adjusted hazard ratio of 2.41 is directionally consistent with the odds ratio of 3.57 reported by Jaradat et al. (7), although the effect size is somewhat attenuated—a pattern commonly observed when biomarkers are tested in independent samples. The discriminative performance of MAR was modest (AUC 0.648) and comparable to that of NLR and SIRI. These findings indicate that MAR alone has insufficient accuracy for individual-level risk prediction and should be interpreted as a supplementary tool rather than a standalone decision-making instrument. This finding is consistent with the broader literature, which has shown that a range of systemic inflammatory indices—including NAR (9), LMR (10, 15), glucose-to-albumin ratio (11), and C-reactive protein-to-albumin ratio (12)—carry prognostic information in ICH and other acute conditions, but that no single index has emerged as clearly dominant. Machine learning approaches that integrate multiple clinical and laboratory variables may ultimately provide superior prognostication in elderly ICH patients (16).

The biological rationale for MAR rests on the integration of two interrelated pathophysiological processes. Monocyte-driven inflammation is central to secondary brain injury after ICH; circulating monocytes are recruited to the perihematomal region, where they contribute to edema propagation and neuronal damage (4). Hypoalbuminemia, widespread in the elderly, reduces colloid osmotic pressure, impairs antioxidant capacity, and has been linked to larger hematoma volumes and increased complication-related mortality in prior work (5, 6). Advanced age and hypoalbuminemia have been specifically identified as risk factors for complication-related mortality beyond 30 days in older ICH patients (6). MAR thus captures a composite “damage–repair” imbalance, a concept further supported by our finding of a weak but significant correlation between MAR and hematoma volume—consistent with the report by Fu et al. that MAR is associated with hematoma expansion (8).

The optimal cutoff derived in this cohort—0.021—is numerically higher than the 0.018 reported by Jaradat et al. (7). This discrepancy has several plausible explanations. First, our cohort was restricted to patients aged ≥65 years, in whom lower baseline albumin and a more pro-inflammatory monocyte phenotype (inflammaging) (17) may shift the MAR distribution upward. Second, differences in albumin measurement methodology (bromocresol green vs. bromocresol purple) can introduce systematic inter-laboratory variation. Third, the Youden index across the candidate cutoffs examined (0.018–0.023) exhibited a broad plateau (Table 2), with values ranging from 0.183 to 0.259. The choice of 0.021 over 0.022 was driven by clinical practicality: although the Youden index was fractionally higher at 0.022 (0.259 vs. 0.258), adopting the higher cutoff would have reduced sensitivity from 57.5 to 51.3%, potentially missing a substantial proportion of high-risk patients. Given the near-equivalence of these two thresholds, we recommend that future studies report the full range of candidate cutoffs and their associated performance characteristics rather than fixating on a single value. This cutoff requires external validation before clinical application and should not be directly extrapolated to other populations.

A key methodological issue concerns the collinearity between GCS and the ICH score, which precluded the inclusion of both in the primary multivariable model. The ICH score is the most widely validated prognostic scale for sICH and integrates age, GCS, hematoma volume, IVH, and infratentorial origin (14). We retained GCS in the primary model because our aim was to evaluate MAR’s independent association after adjusting for the most granular measure of neurological status, while the ICH score was assessed in a dedicated sensitivity analysis. This sensitivity analysis showed that MAR remained significantly associated with mortality after adjustment for the ICH score, although the incremental improvement in discrimination was small (Δ*C* = 0.020). However, the VIF of 6.9 indicates that the coefficients in this sensitivity model may be unstable, and the incremental gain must be interpreted with caution. Regarding incremental utility, NRI, IDI, calibration assessment, and decision curve analysis could not be performed because individual-level predicted probabilities were not available in this retrospective dataset. Future prospective studies with larger sample sizes and lower collinearity should formally assess the added value of MAR over the ICH score using net reclassification improvement or decision curve analysis (18). Until such data are available, MAR should be viewed as a potential adjunct to, rather than a replacement for, established prognostic models.

The clinical appeal of MAR lies in its simplicity and practicality. Complete blood count and albumin are among the first tests ordered upon emergency department arrival, with results typically available within 30–60 min. The ratio can be calculated automatically in the electronic health record and flagged for nursing staff if it exceeds a prespecified threshold. A nursing protocol triggered by an elevated MAR could include intensified neurological monitoring, early nutritional consultation, and prompt escalation to the neurocritical care team. This aligns with the broader trend toward laboratory-informed nursing surveillance in acute stroke care (19). However, given its limited discriminative power, MAR should be used strictly as a supplement to—not a replacement for—established prognostic tools such as the ICH score.

Our study has several limitations. First, the retrospective single-center design limits generalizability. Second, we did not collect data on pre-stroke functional status, frailty indices, or body mass index, which may confound the association between MAR and mortality. Third, only a single admission MAR value was analyzed; dynamic changes during the acute phase may provide additional prognostic information. Fourth, we did not systematically assess functional outcomes, such as the 90-day modified Rankin Scale score. This represents a significant limitation, as recent evidence underscores that even among maximally treated ICH patients, the long-term burden of functional disability remains substantial, and patient-centered outcomes are crucial for evaluating the true impact of the disease (20). Accordingly, our conclusions should be restricted to short-term mortality prediction and should not be extrapolated to broader “prognostic value.” Fifth, the optimal cutoff was derived from the same dataset in which it was tested, and its performance likely overestimates true discriminative ability; independent prospective validation is essential. Sixth, our exclusion of patients with active infection or immunosuppressant use limits applicability to these common clinical scenarios. Seventh, we excluded patients who died within 24 h of admission (*n* = 8). Although this was intended to ensure that MAR was evaluated in patients with a minimum duration of exposure to the post-hemorrhagic inflammatory response, it may have excluded the most severe cases, including those with devastating brainstem hemorrhages or ultra-early neurologic death. As a result, the discriminative performance of MAR reported here (AUC 0.648) may be underestimated relative to its performance in an unselected cohort, and its utility in identifying the most extreme high-risk cases at the moment of admission remains untested. Eighth, we excluded patients with active malignancy or severe primary coagulopathy. This design choice strengthens the internal validity of MAR as a biomarker of ICH-driven pathophysiology by removing major competing risks of death, but it limits generalizability to the broader elderly ICH population, in which these comorbidities are common and prognostically relevant. Ninth, we did not systematically collect data on pre-admission antithrombotic medication use (antiplatelet or anticoagulant agents), which precluded adjustment for this variable in our multivariable models. Anticoagulant-associated ICH may carry a distinct prognosis, and this residual confounding should be addressed in future studies. Tenth, we did not collect data on critical treatment-related factors such as hematoma evacuation, decompressive craniectomy, sedation intensity, mechanical ventilation duration, or nutritional support protocols. These unmeasured interventions likely represent substantial sources of residual confounding, and their absence should be carefully considered when interpreting our findings. Eleventh, patients with active COVID-19 were excluded by the infection criterion, but subclinical or recent SARS-CoV-2 infection could not be systematically ruled out and may have influenced inflammatory indices during the study period. Twelfth, the modest sample size (*n* = 206) with 80 events yielded approximately 16–20 events per variable in the primary multivariable model, which is acceptable but may limit the precision of effect estimates; the wide confidence intervals for AUC and HR should be interpreted accordingly. Thirteenth, the inability to calculate NRI, IDI, calibration metrics, and DCA limits the assessment of incremental clinical utility. Finally, the inability to include the ICH score in the primary multivariable model due to collinearity limits direct comparison with studies that have used this scale, and the incremental value analysis was *post hoc* and should be confirmed in larger cohorts.

## 5 Conclusion

In this independent single-center retrospective cohort of 206 elderly patients with spontaneous intracerebral hemorrhage, admission MAR was independently associated with 30-day all-cause mortality, with a moderate effect size (adjusted HR 2.41) and limited discriminative ability (AUC 0.648). The optimal cutoff of 0.021 differed from the previously reported threshold of 0.018, reflecting cohort- and method-specific variations that underscore the need for external validation. As a simple, routinely available laboratory parameter, MAR may serve as a supplementary—not standalone—bedside tool for early risk stratification. Given the absence of functional outcome data, our findings should be interpreted strictly in the context of short-term mortality prediction. Prospective multicenter studies are required to validate the optimal cutoff, to formally evaluate whether MAR adds incremental value beyond established prediction models such as the ICH score using NRI, IDI, calibration assessment, and decision curve analysis, and to assess the clinical utility of serial MAR measurements.

## Data Availability

The data analyzed in this study is subject to the following licenses/restrictions: the datasets analyzed during the current study are not publicly available due to institutional privacy regulations governing patient health information. These restrictions are imposed by the Medical Ethics Committee of Shanxi Provincial People’s Hospital to protect patient confidentiality. Data are available from the corresponding author upon reasonable request, subject to institutional ethics committee approval. Requests to access these datasets should be directed to Santao Zhang, glh12020025@163.com.
