# Supplementary material for: Predictive value of the monocyte-to-albumin ratio at admission for 30-day mortality in elderly patients with spontaneous intracerebral hemorrhage: a retrospective analysis
Source: Front Neurol. 2026 Sep 30;17:1948936. doi: 10.3389/fneur.2026.1948936 (PMC13661444; doi:10.3389/fneur.2026.1948936)
Supplement: Supplementary file 1 [file Supplementary_file_1.DOCX]

**Supplementary Material**

Predictive Value of the Monocyte-to-Albumin Ratio at Admission for 30-Day Mortality in Elderly Patients with Spontaneous Intracerebral Hemorrhage: A Retrospective AnalysisLihui Gong, Fenglin Liu, Keyi Zhao, Haoshuang Zhao, Sujun Guo, Santao Zhang*

**Supplementary Figure S1**

STROBE flow diagram illustrating the screening and inclusion process. Of 268 patients assessed for eligibility, 62 were excluded (secondary ICH, 18; active infection, 14; immunosuppressant use, 10; end‑stage organ disease or active malignancy, 8; death within 24 hours of admission, 8; missing laboratory data, 4), leaving 206 patients in the final analysis. Of these 206 patients, 11 died between 24 and 48 hours, and an additional 69 died between 48 hours and 30 days (total 30‑day mortality: 80/206, 38.8%).

**Supplementary Figure S2**

Scatter plot showing the relationship between admission MAR and baseline hematoma volume. The solid red line represents the linear regression fit (standardized β = 0.20, P = 0.004). Spearman ρ = 0.23 (P < 0.001). The gray shaded area indicates the 95% confidence band.

**Supplementary Table S1.** Baseline characteristics stratified by the optimal MAR cutoff (0.021).

| **Characteristic** | **MAR ≤ 0.021 (n=120)** | **MAR > 0.021 (n=86)** | **P‑value** |
| --- | --- | --- | --- |
| Age, years, median (IQR) | 73 (68–79) | 76 (71–82) | 0.011 |
| Male sex, n (%) | 65 (54.2) | 45 (52.3) | 0.779 |
| Hypertension, n (%) | 98 (81.7) | 70 (81.4) | 0.931 |
| Diabetes mellitus, n (%) | 33 (27.5) | 25 (29.1) | 0.754 |
| Atrial fibrillation, n (%) | 13 (10.8) | 11 (12.8) | 0.671 |
| Prior stroke, n (%) | 27 (22.5) | 23 (26.7) | 0.485 |
| GCS score, median (IQR) | 14 (11–15) | 9 (6–13) | <0.001 |
| Hematoma volume, mL, median (IQR) | 17.0 (8.8–30.5) | 43.5 (28.0–66.0) | <0.001 |
| IVH, n (%) | 19 (15.8) | 35 (40.7) | <0.001 |
| Infratentorial, n (%) | 5 (4.2) | 9 (10.5) | 0.088 |
| ICH score, median (IQR) | 1 (0–1) | 2 (1–3) | <0.001 |
| Monocyte, ×10⁹/L, median (IQR) | 0.48 (0.36–0.62) | 0.82 (0.66–1.08) | <0.001 |
| Albumin, g/L, median (IQR) | 38.2 (35.0–41.8) | 30.8 (27.0–34.0) | <0.001 |
| NLR, median (IQR) | 3.40 (2.25–5.20) | 7.10 (5.00–11.80) | <0.001 |
| SIRI, median (IQR) | 1.52 (0.85–2.85) | 6.00 (4.00–11.20) | <0.001 |
| 30‑day mortality, n (%) | 34 (28.3) | 46 (53.5) | <0.001 |

IQR, interquartile range; GCS, Glasgow Coma Scale; IVH, intraventricular hemorrhage; NLR, neutrophil‑to‑lymphocyte ratio; SIRI, systemic inflammatory response index.

**Supplementary Table S2.** Multivariable Cox regression model including the ICH score (sensitivity analysis)

| **Variable** | **Adjusted HR (95% CI)** | **P‑value** |
| --- | --- | --- |
| MAR >0.021 | 2.02 (1.25–3.26) | 0.004 |
| ICH score (per point) | 1.38 (1.09–1.74) | 0.007 |
| Age, per year | 1.00 (0.97–1.04) | 0.892 |
| Hematoma volume, per mL | 1.01 (1.00–1.02) | 0.142 |

**Note:** The model included the MAR group, ICH score (continuous), age, and hematoma volume. The GCS and IVH were omitted to avoid redundancy with the ICH score components; hematoma volume was retained as a continuous variable to capture its independent dose-response effect. The variance inflation factor (VIF) for the ICH score was 6.9, indicating moderate collinearity. The C‑statistic of the baseline model incorporating the ICH score alone was 0.731; the addition of MAR resulted in a C‑statistic of 0.751, demonstrating a small incremental improvement in model discrimination.

**Supplementary Table S3.** Sensitivity analysis: multivariable Cox regression for 30-day mortality after excluding patients who died between 24 and 48 hours (n=195)

| **Variable** | **Adjusted HR (95% CI)** | **P‑value** |
| --- | --- | --- |
| MAR >0.021 (vs. ≤0.021) | 2.18 (1.29–3.68) | 0.003 |
| Age, per year | 1.01 (0.98–1.05) | 0.518 |
| GCS, per point | 0.89 (0.83–0.96) | 0.003 |
| Hematoma volume, per mL | 1.02 (1.01–1.03) | <0.001 |
| IVH, yes vs. no | 1.10 (0.63–1.92) | 0.738 |

Among the 206 patients in the final cohort, 11 died between 24 and 48 hours after admission. These patients were excluded in this sensitivity analysis, leaving 195 patients. Combined with the 8 patients who died within 24 hours (already excluded from the main cohort by criterion 6), this sensitivity analysis effectively tests MAR's predictive performance in patients who survived the first 48 hours. The proportional hazards assumption held for all variables (Schoenfeld residual test P > 0.05).

**Note:** This sensitivity analysis was conducted to address potential reverse‑causation bias, i.e., the possibility that terminal physiological derangements present on admission might confound the association between MAR and mortality. The finding that MAR >0.021 remained a significant independent predictor (adjusted HR 2.18, P=0.003) supports the robustness of the primary analysis.

**Supplementary Table S4.** Predictive performance of MAR, NLR, and SIRI for 90-day mortality.

| **Index** | **AUC (95% CI)** | **Optimal Cutoff (90‑day)** | **Adjusted HR (95% CI)*** | **P‑value** |
| --- | --- | --- | --- | --- |
| MAR | 0.631 (0.556–0.706) | 0.021 | 2.05 (1.33–3.16) | 0.001 |
| NLR | 0.626 (0.550–0.702) | 5.60 | 1.74 (1.12–2.70) | 0.014 |
| SIRI | 0.619 (0.542–0.696) | 3.70 | 1.69 (1.09–2.62) | 0.019 |

*Adjusted hazard ratios from Cox proportional hazards models including age, GCS score, hematoma volume, and intraventricular hemorrhage. The 90‑day mortality rate was 44.7% (92/206). AUC, area under the receiver operating characteristic curve; CI, confidence interval; HR, hazard ratio.

**Note:** MAR demonstrated a consistent, independent association with 90‑day mortality, comparable to NLR and SIRI. The discriminative performance at 90 days (AUC 0.631) was slightly lower than that observed for 30‑day mortality (AUC 0.648 in the main text), likely reflecting the increasing influence of post‑discharge factors on long‑term outcomes.
